# The Pupil as a Guide in the Administration of Chloroform

**Published:** 1888-01

**Authors:** H. J. Nelson


### THE PUPIL AS A GUIDE IN THE ADMINISTRATION OF CHLOROFORM.

It is always difficult to determine the exact moment in which a patient is sufficiently under the influence of chloroform for an operation to be proceeded with. The sign of conjunctival reflex action, which is usually relied upon, is very variable and often misleading. Mr. Henry J. Nelson formulates the following conclusions as a guide in this respect (*British Medical Journal*):

1. The effect produced by chloroform on the pupil is at first dilatation, varying in degree and duration, then contraction as the narcosis becomes profound, and dilatation again when the sensibility is returning. If the administration be still continued, with the pupil strongly contracted and motionless, the pupil will also dilate, but in this case more suddenly and completely, and will be coincident with a state from which it will be difficult or impossible to resuscitate the patient. This latter is the dilatation of asphyxia.

2. So long as the pupil dilates in response to excitation by pinching, etc., the patient is not sufficiently narcotized for the operation to be proceeded with, unless the latter is slight and does not require complete anæsthesia.

3. When the pupil becomes strongly contracted and immobile, no more chloroform should be given until it begins to dilate again. If, then, further anæsthesia be required, a little more chloroform should be given till the pupil again contracts.

4. The occurrence of sickness causes dilatation similar to, but more sudden than, that which happens when sensibility is returning, and the efforts of vomiting have the effect of arousing the patient.

The watching of the respiration and the pulse, which are doubtless the best indications of the effect produced on the individual by chloroform, and, therefore, of vital importance for safe administration, does not in many cases furnish evidence of the state of sensibility, in regard to which he holds the observation of the pupil to be of greatest assistance. The sign usually relied on, namely, the insensibility of the conjunctiva, is by no means a satisfactory test, for in many cases conjunctival anæsthesia is established long before the patient can be said to be under the influence of the drug. By observing the pupil, the administrator of chloroform can tell at once when the effect of the drug is on the wane, because the pupil then begins to dilate slowly. Noticing this, he can, by the administration of a few drops more chloroform till the pupil contracts again, prevent the occurrence of struggling and interruption of the operation. In this way he can keep the patient in the state most suitable for the satisfactory performance of the operation without narcotizing him more than is necessary. The amount of chloroform required to maintain a state of anæsthesia is much less than that required to put a patient under its influence several times, and as it is admittedly a dangerous drug, the less administered the better, especially in operations of long duration. And by allowing the patient partially to recover, one runs the risk of the occurrence of sickness and vomiting, which is always an awkward, and often a dangerous accident. In the absence of such a guide as the observation of the pupil, the chloroform is likely to be given in a rather haphazard way, dosing the patient till narcosis is profound, perhaps too much so, then interrupting the operation till the danger is averted by arousing him, or waiting until signs of feeling, such as struggles or a cry of pain, give indication for more chloroform.

The observation of the pupil also furnishes a fair indication of the effect produced by chloroform, its size bearing a constant relation to the state of the blood pressure. In the experiments on dogs the blood pressure in the carotid artery was recorded on charts, and it was found that contraction only occurred when the pressure had fallen considerably, and, on removal of the chloroform, dilatation only took place when the pressure had risen to a certain height. As reduction of the blood pressure was pronounced by the committee of the British Medical Association to be one of the chief dangers in chloroform administration, the presence of a sign by which the occurrence of that important condition can be recognized must be of practical value.—*The American Medical Digest.*

